# O-RADS MRI to classify adnexal tumors: from clinical problem to daily use

**DOI:** 10.1186/s13244-023-01598-0

**Published:** 2024-01-30

**Authors:** Yohann Dabi, Andrea Rockall, Elisabeth Sadowski, Cyril Touboul, Leo Razakamanantsoa, Isabelle Thomassin-Naggara, the EURAD study group, E. Poncelet, A. Jalaguier-Coudray, A. Guerra, L. S. Fournier, S. Stojanovic, I. Millet, N. Bharwani, V. Juhan, T. M. Cunha, G. Masselli, C. Balleyguier, C. Malhaire, N. Perrot, M. Bazot, P. Taourel, E. Darai, A. G. Rockall

**Affiliations:** 1 APHP, Sorbonne Université, Hôpital Tenon, Service de Gynecologie Et Obstétrique, 75020 Paris, France; 2 Institut Universitaire de Cancérologie, Sorbonne Université, Hôpital Tenon, Service de Radiologie, 58 Avenue Gambetta, 75020 Paris, France; 3 Department of Surgery and Cancer, Faculty of Medicine, Imperial College London, London, UK; 4 Department of Radiology, Imperial College Healthcare NHS Trust, London, UK; 5 Department of Radiology, University of Michigan, Madison, WI USA; 6 APHP, Sorbonne Université, Hôpital Tenon, Service de Radiologie, 58 Avenue Gambetta, 75020 Paris, France

**Keywords:** O-RADS MRI, EURAD study, Adnexal mass, Ultrasonography, Predicting malignancy

## Abstract

Eighteen to 35% of adnexal masses remain non-classified following ultrasonography, leading to unnecessary surgeries and inappropriate management. This finding led to the conclusion that ultrasonography was insufficient to accurately assess adnexal masses and that a standardized MRI criteria could improve these patients’ management. The aim of this work is to present the different steps from the identification of the clinical issue to the daily use of a score and its inclusion in the latest international guidelines. The different steps were the following: (1) preliminary work to formalize the issue, (2) physiopathological analysis and finding dynamic parameters relevant to increase MRI performances, (3) construction and internal validation of a score to predict the nature of the lesion, (4) external multicentric validation (the EURAD study) of the score named O-RADS MRI, and (5) communication and education work to spread its use and inclusion in guidelines. Future steps will include studies at patients’ levels and a cost-efficiency analysis.

**Critical relevance statement **We present translating radiological research into a clinical application based on a step-by-step structured and systematic approach methodology to validate MR imaging for the characterization of adnexal mass with the ultimate step of incorporation in the latest worldwide guidelines of the O-RADS MRI reporting system that allows to distinguish benign from malignant ovarian masses with a sensitivity and specificity higher than 90%.

**Key points**

• The initial diagnostic test accuracy studies show the limitation of a preoperative assessment of adnexal masses using solely ultrasonography.

• The technical developments (DCE/DWI) were investigated with the value of dynamic MRI to accurately predict the nature of benign or malignant lesions to improve management.

• The first developing score named ADNEX MR Score was constructed using multiple easily assessed criteria on MRI to classify indeterminate adnexal lesions following ultrasonography.

• The multicentric adnexal study externally validated the score creating the O-RADS MR score and leading to its inclusion for daily use in international guidelines.

## Introduction

An accurate discrimination between benign and malignant ovarian lesions is of paramount importance in gynecologic management. The precise characterization of adnexal lesions can significantly impact patient care and treatment outcomes. Indeed, accurate characterization helps prevent unnecessary surgical interventions in cases where the lesion is determined to be benign [1, 2]. Unwarranted surgeries can lead to increased morbidity, prolonged hospital stays, and higher healthcare costs. For women of reproductive age, preserving fertility is a critical concern. Accurate characterization allows clinicians to make informed decisions about fertility-sparing treatments when dealing with benign lesions, thereby safeguarding the patient’s ability to conceive in the future [3, 4]. For malignant lesions, precise characterization contributes to the determination of the optimal treatment approach. Discussion in multidisciplinary team sessions helps gynecologic oncologists plan appropriate surgical procedures, chemotherapy, or radiation therapy, leading to better treatment outcomes. Early detection and referral of malignant lesions to gynecologic oncologists contribute to improved survival rates [5]. Earlier surgical removal and pathological analysis lead to more effective treatments and better chances of disease control. Prompt and accurate diagnosis can help alleviate patient anxiety and emotional distress associated with uncertainty about their condition. Providing patients with clear information and appropriate referrals instills confidence in their healthcare providers [6]. Accurate characterization allows for efficient coordination among various medical specialties, such as gynecologists, radiologists, pathologists, and oncologists, to provide comprehensive and patient-centered care. Patients can make well-informed decisions about their treatment options when they have a clear understanding of their diagnosis and prognosis.

To achieve these benefits, continuous efforts should be made to improve diagnostic imaging techniques. The historical reliance on ultrasound as the primary imaging modality for diagnosing ovarian lesions has led to several challenges and limitations in accurately distinguishing between benign and malignant tumors. Among those, factors such as patient characteristics, lesion characteristics, operator expertise, and invasive nature of surgery are important [7, 8]. MR imaging was proven to be an accurate second-line technique many decades ago but was not integrated into standardized clinical protocols and guidelines for adnexal lesion management. The development of a multiparametric approach based on morphological and functional MR criteria allowed to develop a score that strongly increases the negative predictive value of malignancy and thus placed MRI as a useful tool for the management of patients.

In this paper, we will present translating radiological research into a clinical application based on a step-by-step structured and systematic approach methodology. The aim was to validate MR imaging for the characterization of adnexal mass and the ultimate step of incorporation in the latest worldwide guidelines of the O-RADS MRI reporting system. This O-RADS MRI score enables to distinguish benign ovarian masses from malignant ones with a sensitivity and specificity higher than 90% [9, 10]. Moreover, the different ongoing research and professional development of healthcare providers will be presented as they are determinants to ensure that the latest evidence-based practices be included in clinical care.

## Study design and methodology

The O-RADS MRI score development is a model of a top-down approach according to evidence-based medicine [11–14]. The top-down approach involves academic centers and experts while the bottom-up approach involves physicians in daily practice. Each step that led to the completion of the EURAD study (which results allow the princeps publication of O-RADS MRI score) was performed with respect to the scientific method [15].

### Clinical question

The lack of a standardized tool (prior to the validation of the O-RADS MRI score) for predicting malignancy in adnexal masses is a common challenge during weekly dedicated multidisciplinary team (MDT) sessions, limiting collaboration between radiologists and gynecological surgeons. The conventional “subjective” description of adnexal mass on MR imaging to predict malignancy are hardly reproducible across observers. Moreover, the intraoperative frozen section has limits to provide a reliable diagnosis, reinforcing the need for precise preoperative imaging [16].

### Road map

The unmet clinical need refers to the challenge of accurately identifying malignant (cancerous) adnexal masses during ultrasound (US) examinations. Adnexal masses detected through US examinations can appear indeterminate, making it difficult for clinicians to confidently determine whether they are benign (non-cancerous) or malignant [17]. As a result, there is a risk of false-positive cases, where patients might undergo unnecessary cancer surgeries due to the uncertainty surrounding the diagnosis [2, 18].

To address this issue, researchers have conducted observational studies, particularly single-center diagnostic test accuracy studies, to assess the performance of different imaging techniques in distinguishing between benign and malignant adnexal masses [19–21]. MRI is known for its excellent soft tissue contrast and ability to provide detailed images of internal organs, making it a valuable tool in diagnosing adnexal masses [22]. In 2005, Kinkel et al. conducted a Bayesian analysis to evaluate the incremental benefit of using a second imaging test (such as MRI) after an inconclusive US examination. Bayesian analysis is a statistical approach that can be used to combine prior knowledge (prior probability) with new evidence (likelihood) to update the probability of an event (posterior probability). This paper concluded that in women with an indeterminate ovarian mass at US, MR imaging results contributed to a change in the probability of ovarian cancer in both pre- and postmenopausal women more than did CT or combined gray-scale and Doppler US results [23]. Based on these elements, the European Society of Urogenital Radiology wrote the guidelines for MR imaging of the sonographically indeterminate adnexal mass and proposed a first algorithmic and problem-solving approach based on signal characteristics and morphology [24].

Overall, these studies and analyses aim to improve the diagnostic accuracy in distinguishing benign from malignant adnexal masses, thereby reducing the number of false-positive cases. However, these studies included only morphological criteria and did not reach enough negative predictive value to really impact the number of unnecessary cancer surgeries.

In this setting, strong and reproducible functional MR criteria were developed to improve adnexal mass characterization. The ability of DCE MR imaging was first proven to improve the evaluation of the origin of purely solid ovarian masses which remains the first step to analyze a pelvic mass [25]. In this paper, the DCE MR enhancement rate was higher for uterine leiomyomas than for ovarian fibromas in terms of both maximal enhancement (*p* < 0.001) and enhancement rate at 30 s (*p* = 0.009), 60 s (*p* = 0.007), and 90 s (*p* = 0.0009) [25].

Then, testing of technical developments was initiated in a Ph.D. to pursue the usefulness of DCE MR to characterize solid tissue in adnexal masses. First, Thomassin-Naggara et al. proved that the DCE MR criteria of the time-intensity curve (enhancement amplitude, time to enhancement, maximal slope) were correlated with angiogenesis biomarkers, i.e., pericyte coverage index and VEGF receptors expression [25]. A second series of papers demonstrate the feasibility and the value of functional MR imaging to discriminate benign from malignant lesions [26–28]. In addition, these papers highlighted that functional criteria could improve diagnostic value when combined with morphological criteria (25% for DCE/15% for DWI of diagnosis correctly reclassified).

Based on the literature between 2002 and 2012, a systematic review published by gynecologists established pelvic MRI as the “gold standard” in the subsequent evaluation of US indeterminate adnexal lesions. In this paper, the authors concluded that MRI with intravenous (IV) contrast administration provides the highest post-test probability of ovarian cancer detection. However, the preponderant contribution of MRI in adnexal mass evaluation is its specificity because it provides a confident diagnosis of many benign adnexal lesions [29].

Despite the amount of evidence in the literature, at that time, there was no translation into clinical practice and the need for standardization of the model of the RADS system to have an impact on the management of patients existed. Hence, in 2013, a new study reported the ability to combine the MR criteria in a multivariate analysis and build the first version of the score named ADNEX MR score [30]. This score was developed on a monocentric cohort from a referral tertiary care center for gynecological malignancies on masses considered as indeterminate at ultrasonography (US). The study population comprised 394 women who underwent MR imaging between January 1, 2008, and October 30, 2010, for the characterization of 497 adnexal masses that were seen in US. Then, masses were chronologically divided into a training set (329 masses) and a validating set (168 masses). The score was accurate and reproducible in this retrospective cohort.

Several teams in the world validated in different monocentric studies this score [31–33]. Based on the amount of retrospective data, and the necessary prior clinical use of an external validation of this new scoring system, a prospective multicentric European cohort was launched: the EURAD Study [34]. This work was funded by a grant from the Société d’Imagerie de la Femme (SIFEM) and supported by the National Institute of Health Research Imperial Biomedical Research Centre and the Cancer Research UK Imperial Centre.

**Fig. 1:**
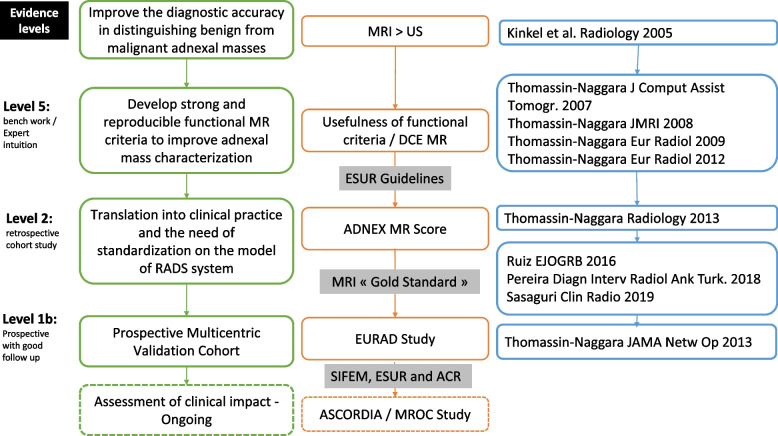
Evidence levels, steps of progression, and publications associated

**Fig. 2:**
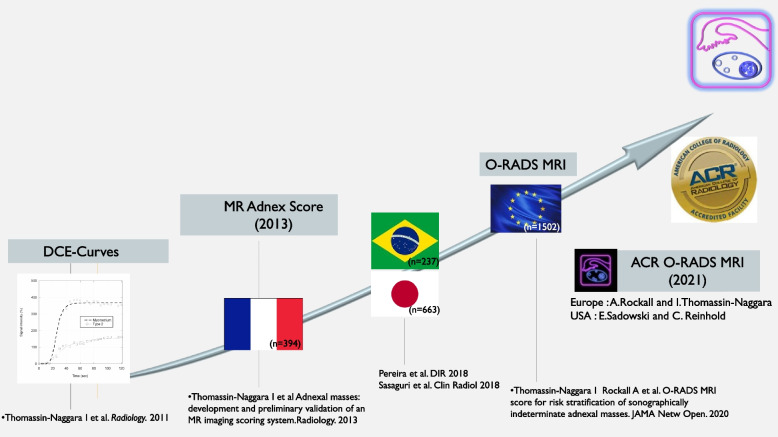
Validation of functional imaging to improve adnexal masses characterization

A summary of the road map that led to the EURAD study is presented in Figs. 1 and 2.

### EURAD Study

During the European Congress of Urogenital Radiology in Dubrovnik in 2011 [35], 15 expert radiologists agreed to participate in a multicentric validation of the ADNEX MR score.

Two investigator coordinators were designated to write the protocol which was discussed with all investigators who were key opinion leaders in adnexal mass imaging in Europa.

#### Study design

The EURAD Study was a prospective multicenter study finally conducted between March 1, 2013, and March 31, 2018. Participant enrollment took place between March 1, 2013, and March 31, 2016.

#### Inclusion criteria

Recruitment was undertaken in 15 centers, each with a main investigator from the European Society of Urogenital Radiology Female Pelvic Imaging working group or from Société d’Imagerie de la Femme. Several studies underlined that 18 to 31% of ovarian tumors remain indeterminate after ultrasonography [7, 8]. For the EURAD Study, the main inclusion criteria were the description at ultrasonography of an indeterminate mass at ultrasonography without using any ultrasonographic scores. This is in line with clinical routine. Furthermore, the subjective analysis of adnexal masses at ultrasonography by an expert is proven more accurate than any ultrasonographic score [36]. In order to ensure the notion of an expert sonographer, the quality of ultrasonography was quoted in addition, with a quality score of 7 points for all patients included in the EURAD Study.

#### MR imaging protocol

Patients underwent a routine pelvic MR imaging (1.5 T or 3 T), including morphological sequences (T2, T1 with and without fat suppression, and T1 after dynamic gadolinium injection) and functional sequences (perfusion and diffusion-weighted sequences).

#### Data collection

Prospectively, one senior (expertise in pelvic MR imaging > 5 years) and one junior radiologists (expertise in pelvic MR imaging 6–12 months) independently analyzed the different MR criteria to characterize adnexal masses. The MR report was issued as standard, and the patients were managed accordingly. Then, the reader classified the mass using the score.

#### Reference standard

The classification was compared to routine clinical management which can include surgical procedures and histology or standard clinical follow-up depending on the most appropriate routine practice. Finally, 362 of 1340 patients (27.0%) undergoing expectant management with a 2-year follow-up, which was completed by March 31, 2018. The decision to not exclude adnexal masses without surgery and only expectant management was crucial to have a prevalence of malignancy similar to the clinical routine.

#### Data collection

##### Preliminary period

A session of 30 DICOM cases was downloaded for a session for all teams participating to the multicenter validation to evaluate the quality of the MR examination. Moreover, a training session for all investigators was conducted during the ESUR Congress in Edinburg in 2012 [37].

##### Prospective data collection

The readers were informed of clinical and ultrasonographic data, analyzed the different MR criteria present in the MR Score, and classified the mass. At the end of the procedure, DICOM data was sent to the center coordinator for another reading of each case will be performed by two other senior radiologists without any knowledge of clinical, ultrasonographic, pathological, or follow-up data.

The reproducibility of the classification was tested between the junior and the senior radiologist. After anonymization, images were analyzed by another senior radiologist of another center blinded from any clinical or ultrasonographical data at the end of the study and correlated with the reference standard.

#### Data analysis

Data analysis was performed by a senior statistician under clinical guidance to better apprehend the clinical aspects associated with the score analysis.

#### Main results

The MR score was accurate when stratifying the risk of malignancy in adnexal masses with a sensitivity of 0.93 and a specificity of 0.91, reproducible with a high interrater agreement among both experienced and junior readers (*κ* = 0.784), and able to correctly reclassify the mass origin as non-adnexal with a sensitivity of 0.99 a specificity of 0.78. These results were published in the journal *JAMA Network Open* under the name O-RADS MRI score (that replaced “ADNEX MRI” name), and this publication is considered as the princeps publication of the score [38].

## Impact

### Clinical application development

Regarding the impact, the development of O-RADS MRI score has standardized MR protocol acquisition including gadolinium injection and functional DCE and DW MR sequences in European countries. Moreover, a standardized report was built and diffused by the SIFEM, ESUR, and ACR. An update of ESUR Recommendations for MR imaging of the sonographically indeterminate adnexal mass was published integrating functional criteria in MR protocol [39]. The American College of Radiology and the European Society of Radiology endorsed a common lexicon to describe adnexal masses at MR imaging [40].

The use of MR score helps in improving patient management selecting women who would benefit from a referral to specialized multidisciplinary center for ovarian cancer [6]. In France, a patient with an adnexal mass-rated O-RADS MRI 4 or 5 should be referred to an accredited center defined by a minimal number of advanced ovarian cancer surgeries of 20 per year, as many studies demonstrated a correlation between the survival of the patient and the expertise of the surgeon and his multidisciplinary team.

Based on the EURAD Study’s findings, the guidelines were developed for incorporating MR imaging into the clinical management of adnexal masses. In 2019, French guidelines clearly outline when and how to use MR scores and recommend to include a score at the end of any MR report for the characterization of adnexal masses [9]. Subsequently, international societies such as the American College of Radiology (ACR) and the European Society of Radiology (ESR) included the O-RADS MRI Scoring system in the guidelines for the diagnostic workup of indeterminate adnexal masses following ultrasonography [10, 41]. Compared with other RADS systems (BI-RADS, LI-RADS, GI-RADS), the O-RADS MR score was based on a statistical analysis and tested in a clinical outline. Moreover, few criteria are needed to be learned, and the success of this classification was its easiness of use for non-specialized radiologists, as the reproducibility of the score was well demonstrated in many different clinical studies [30, 38]. Some authors reported on its implementation in clinical practice [42, 43].

### Education and training

A determinant factor to enable the adoption of this new system is a large communication campaign. Several educational papers were published since the creation of the O-RADS MRI Score in 2020 [44–46]. Moreover, an educational group of experts was created by the ACR who organized many training programs and workshops around the world to disseminate the knowledge effectively. This group also allows the translation of O-RADS MRI scores in more than ten different languages. Thus, a lot of educational webinars and sessions during the international congress were organized in RSNA ECR, SAR, and ESUR. Many educational resources are available on a dedicated website.

### Clinical implementation and monitoring

Monitoring the impact and gathering feedback are necessary adjustments based on real-world experiences and advancements in technology. In this setting, a clinical trial was initiated in 2018 named ASCORDIA to evaluate the impact of the O-RADS MRI Score on surgical management [47]. Another prospective study named MROC study conducted in the UK also tested the implementation of MR score in a randomized study on the management of patients [48]. This study evaluates the possibility of mpMRI, including O-RADS MR Score, providing an improved radiological assessment for the classification and delineation of the extent of disease for patients with suspected ovarian cancer compared to standard of care CT assessment, potentially facilitating more accurate decisions regarding patient management by the MDT. The results of these two prospective studies are not yet published.

### Continuous research and improvement

Different secondary studies were subsequently published following the princeps publication in *JAMA* in 2020 [38]. First, an analysis of misclassified cases using an O-RADS MRI score was performed [49]. The objective was to determine the presumptive causes of these misclassifications which were mainly due to the interpretation of solid tissue or incorrect assignment of mass origin. This publication allowed us to focus on these points’ educational programs. A second study evaluated the necessity to use time-intensity curve in O-RADS MRI Score, which may not be universally available [50]. This study demonstrated that time-intensity curve analysis was more accurate than visual assessment for achieving optimal diagnostic accuracy with the Ovarian-Adnexal Reporting and Data System MRI score. A third study analyzed the impact of the ADC value of the cystic component to improve the performance of the O-RADS MRI score and demonstrated its added value for subcategorizing O-RADS MRI score 4 [51]. Many other studies are ongoing across the world.

### Publication and communication

A metanalysis has been recently published by an Italian team confirming a sensitivity and specificity higher than 90% of the O-RADS MR score in a cohort of 3731 women (4520 adnexal lesions) [52].

To promote the diffusion of the score, an online calculator was developed (https://www.oradsmricalc.com/) [53] to help physicians identify the criteria required to classify. This will contribute to the dissemination of knowledge and favor adoption by the wider medical community. In this setting, a dedicated Twitter account was created to diffuse the new research findings and outcomes through peer-reviewed publications and presentations at relevant conferences.

### Lesson learned

A successful translation of radiological research into a clinical application requires a collaborative effort among researchers, clinicians, and other stakeholders to ensure its successful integration into clinical practice. More than 10 years passed since the publication of the first MR score in 2013 [30]. Morris et al. published a review of the literature describing and quantifying time lags in the health research translation process [54]. Their main conclusion is that 17 years are usually required for research evidence to reach clinical practice [55, 56] but depends on the field investigated. If we analyzed the classical 6 levels of hierarchy for studies on diagnostic tests [57], MR imaging for the characterization of adnexal masses has passed step 1 (validation of technical performance), step 2 (validation of diagnostic performance), and step 3 (validation of diagnostic impact). ASCORDIA and MROC studies will probably help to pass step 4 (validation of therapeutic impact) and more lately step 5 (patient outcomes). However, the validation of step 6 (societal impact) is not yet planned. These elements are summarized in Fig. 2.

In conclusion, challenges along the translational process are multiple, even following guidelines of publications [58–60]. Physician-level barriers include knowing that guidelines exist, knowing or agreeing with their content, and having the time to apply the guidelines in the clinical setting. One of the strengths of the EURAD study was to involve a community of key opinion leaders in different European and American countries that have influenced progressively the clinical practice. Further studies must be conducted to reach the complete translation in clinical routine.

## Data Availability

Not applicable.
